# 2-Cyano­anilinium dihydrogen phosphate

**DOI:** 10.1107/S1600536809034898

**Published:** 2009-09-12

**Authors:** Li Zhang

**Affiliations:** a Ordered Matter Science Research Center, College of Chemistry and Chemical Engineering, Southeast University, Nanjing 210096, People’s Republic of China

## Abstract

In the cation of the title compound, C_7_H_7_N_2_^+^·H_2_PO_4_^−^, the nitrile group and the benzene ring are almost coplanar (r.m.s. deviation = 0.0035 Å). The cations and anions are connected by inter­molecular N—H⋯O, O—H⋯O and O—H⋯N hydrogen bonds, together with π–π inter­actions [centroid–centroid distance = 3.8131 (9) Å], forming a three-dimensional network.

## Related literature

For applications of metal-organic coordination compounds, see: Fu *et al.* (2007 ▶); Chen *et al.* (2000 ▶); Fu & Xiong (2008 ▶); Xiong *et al.* (1999 ▶); Xie *et al.* (2003 ▶); Zhang *et al.* (2001 ▶). For nitrile derivatives, see: Fu *et al.* (2008 ▶); Wang *et al.* 2002 ▶.
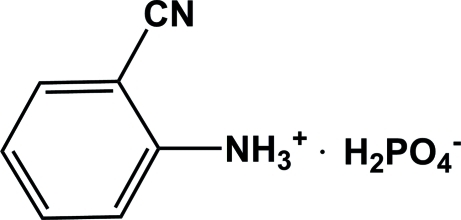


## Experimental

### 

#### Crystal data

C_7_H_7_N_2_^+^·H_2_PO_4_^−^
*M* *_r_* = 216.13
Triclinic, 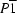

*a* = 6.1471 (12) Å
*b* = 9.3192 (19) Å
*c* = 9.3295 (19) Å
α = 117.20 (2)°
β = 93.75 (2)°
γ = 99.61 (2)°
*V* = 462.51 (16) Å^3^
*Z* = 2
Mo *K*α radiation
μ = 0.29 mm^−1^
*T* = 298 K
0.30 × 0.25 × 0.20 mm

#### Data collection

Rigaku Mercury2 diffractometer
Absorption correction: multi-scan (*CrystalClear*; Rigaku, 2005 ▶) *T*_min_ = 0.94, *T*_max_ = 1.00
4831 measured reflections
2110 independent reflections
1852 reflections with *I* > 2σ(*I*)
*R*_int_ = 0.030

#### Refinement

*R*[*F* ^2^ > 2σ(*F* ^2^)] = 0.040
*wR*(*F* ^2^) = 0.102
*S* = 1.08
2110 reflections
129 parameters
H-atom parameters constrained
Δρ_max_ = 0.33 e Å^−3^
Δρ_min_ = −0.37 e Å^−3^

### 

Data collection: *CrystalClear* (Rigaku, 2005 ▶); cell refinement: *CrystalClear*; data reduction: *CrystalClear*; program(s) used to solve structure: *SHELXS97* (Sheldrick, 2008 ▶); program(s) used to refine structure: *SHELXL97* (Sheldrick, 2008 ▶); molecular graphics: *SHELXTL* (Sheldrick, 2008 ▶); software used to prepare material for publication: *SHELXTL*.

## Supplementary Material

Crystal structure: contains datablocks I, global. DOI: 10.1107/S1600536809034898/pv2201sup1.cif

Structure factors: contains datablocks I. DOI: 10.1107/S1600536809034898/pv2201Isup2.hkl

Additional supplementary materials:  crystallographic information; 3D view; checkCIF report

## Figures and Tables

**Table 1:** Hydrogen-bond geometry (Å, °)

*D*—H⋯*A*	*D*—H	H⋯*A*	*D*⋯*A*	*D*—H⋯*A*
N1—H1*B*⋯O4^i^	0.89	1.93	2.819 (2)	176
N1—H1*C*⋯O1^ii^	0.89	1.85	2.723 (2)	166
N1—H1*A*⋯O1^iii^	0.89	1.87	2.730 (2)	161
O3—H3*A*⋯N2^iv^	0.82	1.98	2.797 (2)	176
O2—H2*A*⋯O4^v^	0.82	1.76	2.574 (2)	172

Symmetry codes: (i) 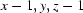; (ii) 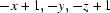; (iii) 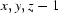; (iv) 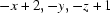; (v) 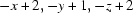.

**Fig. 1.**
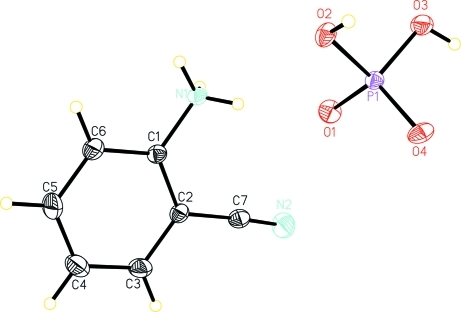
A view of the title compound with the atomic numbering scheme. Displacement ellipsoids are drawn at the 30% probability level.

**Fig. 2.**
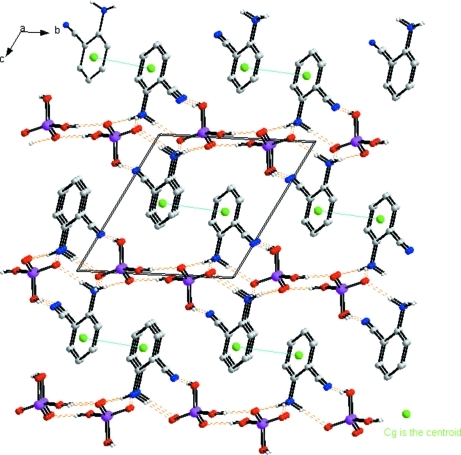
The crystal packing of the title compound, viewed along the a axis showing the hydrogen bonds and the π–π interactions in the title compound. H atoms not involved in hydrogen bonding (dashed lines) have been omitted for clarity

## supplementary crystallographic information

### Comment

The construction of metal-organic coordination compounds has attracted much attention owing to potential functions, such as permittivity, fluorescence, magnetism and optical properties (Fu *et al.*, 2007; Chen *et al.*, 2000; Fu & Xiong (2008); Xie *et al.*, 2003; Zhang *et al.*,2001; Xiong *et al.*, 1999). Nitrile derivatives are a class of excellent ligands for the construction of novel metal-organic frameworks. (Wang *et al.* 2002; Fu *et al.*, 2008). We report here the crystal structure of the title compound, 2-cyanoanilinium dihydrogen phosphate .

In the 2-cyanoanilinium cation (Fig.1), the nitrile group and the benzene ring are almost coplanar. The nitrile group C7≡N2 bond length of 1.137 (3) Å is within the normal range.

In the crystal structure, all the amine group H atoms and H_2_PO_4_^-^ H atoms are involved in N—H···O, O—H···O and O—H···N hydrogen bonds (Table 1) with N atoms of nitrile group and O atoms of H_2_PO_4_^-^ anion. The benzene rings [Cg···Cg] of neighbouring cation systems are separated by 3.8131 (9) Å [Cg is the centroid of the benzene rings]. These hydrogen bonds and π–π interactions link the ionic units into a three-dimensional network (Fig. 2).

### Experimental

The commercial 2-aminobenzonitrile (3 mmol, 0.55 g) and H_3_PO_4_ (0.5 ml) were dissolved in ethanol (20 ml). Colourless block-shaped crystals of the title compound suitable for X-ray analysis were obtained by slow evaporation at room temperature.

### Refinement

All H atoms attached to C atoms were positioned geometrically and treated as riding, with C-H = 0.93 Å and *U*_iso_(H) = 1.2U_eq_(C). The H atoms of H_2_PO_4_^-^ anion and amine group were located in difference Fourier maps and the last stage of refinement they were treated as riding on the O atoms and N atoms, with *U*_iso_(H) = 1.5*U*_eq_(O and N).

### Crystal data

**Table:**

C_7_H_7_N_2_^+^·H_2_PO_4_^−^	*Z* = 2
*M**_r_* = 216.13	*F*(000) = 224
Triclinic, *P*1	*D*_x_ = 1.552 Mg m^−^^3^
Hall symbol: -P 1	Mo *K*α radiation, λ = 0.71073 Å
*a* = 6.1471 (12) Å	Cell parameters from 1852 reflections
*b* = 9.3192 (19) Å	θ = 3.4–27.5°
*c* = 9.3295 (19) Å	µ = 0.29 mm^−^^1^
α = 117.20 (2)°	*T* = 298 K
β = 93.75 (2)°	Block, colourless
γ = 99.61 (2)°	0.30 × 0.25 × 0.20 mm
*V* = 462.51 (16) Å^3^	

### Data collection

**Table:**

Rigaku Mercury2 diffractometer	2110 independent reflections
Radiation source: fine-focus sealed tube	1852 reflections with *I* > 2σ(*I*)
graphite	*R*_int_ = 0.030
Detector resolution: 13.6612 pixels mm^-1^	θ_max_ = 27.5°, θ_min_ = 3.4°
CCD profile fitting scans	*h* = −7→7
Absorption correction: multi-scan (*CrystalClear*; Rigaku, 2005)	*k* = −12→12
*T*_min_ = 0.94, *T*_max_ = 1.00	*l* = −12→12
4831 measured reflections	

### Refinement

**Table:**

Refinement on *F*^2^	Secondary atom site location: difference Fourier map
Least-squares matrix: full	Hydrogen site location: inferred from neighbouring sites
*R*[*F*^2^ > 2σ(*F*^2^)] = 0.040	H-atom parameters constrained
*wR*(*F*^2^) = 0.102	*w* = 1/[σ^2^(*F*_o_^2^) + (0.0436*P*)^2^ + 0.23*P*] where *P* = (*F*_o_^2^ + 2*F*_c_^2^)/3
*S* = 1.08	(Δ/σ)_max_ < 0.001
2110 reflections	Δρ_max_ = 0.33 e Å^−^^3^
129 parameters	Δρ_min_ = −0.37 e Å^−^^3^
0 restraints	Extinction correction: *SHELXL97* (Sheldrick, 2008), Fc^*^=kFc[1+0.001xFc^2^λ^3^/sin(2θ)]^-1/4^
Primary atom site location: structure-invariant direct methods	Extinction coefficient: 0.126 (9)

### Special details

**Table:**

Geometry. All esds (except the esd in the dihedral angle between two l.s. planes) are estimated using the full covariance matrix. The cell esds are taken into account individually in the estimation of esds in distances, angles and torsion angles; correlations between esds in cell parameters are only used when they are defined by crystal symmetry. An approximate (isotropic) treatment of cell esds is used for estimating esds involving l.s. planes.
Refinement. Refinement of F^2^ against ALL reflections. The weighted R-factor wR and goodness of fit S are based on F^2^, conventional R-factors R are based on F, with F set to zero for negative F^2^. The threshold expression of F^2^ > 2sigma(F^2^) is used only for calculating R-factors(gt) etc. and is not relevant to the choice of reflections for refinement. R-factors based on F^2^ are statistically about twice as large as those based on F, and R- factors based on ALL data will be even larger.

### Fractional atomic coordinates and isotropic or equivalent isotropic displacement parameters (Å^2^)

**Table:**

	*x*	*y*	*z*	*U*_iso_*/*U*_eq_	
N1	0.3606 (3)	0.17867 (19)	0.13975 (19)	0.0252 (3)	
H1A	0.4996	0.1956	0.1197	0.038*	
H1B	0.2813	0.2336	0.1078	0.038*	
H1C	0.2980	0.0710	0.0852	0.038*	
C2	0.5068 (3)	0.1896 (2)	0.3985 (2)	0.0275 (4)	
C1	0.3654 (3)	0.2371 (2)	0.3135 (2)	0.0254 (4)	
N2	0.7647 (4)	−0.0074 (3)	0.2631 (3)	0.0524 (6)	
C7	0.6495 (4)	0.0801 (3)	0.3194 (3)	0.0343 (5)	
C3	0.5080 (4)	0.2455 (3)	0.5646 (3)	0.0381 (5)	
H3	0.6040	0.2152	0.6217	0.046*	
C6	0.2255 (4)	0.3362 (3)	0.3937 (3)	0.0406 (5)	
H6	0.1296	0.3674	0.3374	0.049*	
C5	0.2268 (5)	0.3900 (3)	0.5589 (3)	0.0524 (7)	
H5	0.1312	0.4571	0.6128	0.063*	
C4	0.3683 (5)	0.3452 (3)	0.6444 (3)	0.0479 (6)	
H4	0.3687	0.3824	0.7554	0.057*	
P1	0.87212 (8)	0.26924 (6)	0.96817 (6)	0.02198 (17)	
O4	1.1050 (2)	0.36166 (16)	1.05571 (18)	0.0311 (3)	
O3	0.8776 (2)	0.16546 (19)	0.77991 (17)	0.0371 (4)	
H3A	0.9862	0.1229	0.7672	0.056*	
O2	0.7151 (2)	0.38600 (16)	0.97509 (17)	0.0294 (3)	
H2A	0.7833	0.4620	0.9628	0.044*	
O1	0.7610 (2)	0.15769 (17)	1.02994 (18)	0.0334 (4)	

### Atomic displacement parameters (Å^2^)

**Table:**

	*U*^11^	*U*^22^	*U*^33^	*U*^12^	*U*^13^	*U*^23^
N1	0.0267 (8)	0.0251 (8)	0.0264 (8)	0.0079 (6)	0.0025 (6)	0.0141 (6)
C2	0.0282 (10)	0.0272 (9)	0.0308 (10)	0.0111 (8)	0.0046 (8)	0.0151 (8)
C1	0.0274 (10)	0.0233 (9)	0.0269 (10)	0.0076 (7)	0.0031 (7)	0.0128 (8)
N2	0.0607 (14)	0.0626 (14)	0.0557 (13)	0.0420 (12)	0.0216 (11)	0.0354 (11)
C7	0.0382 (11)	0.0409 (12)	0.0354 (11)	0.0170 (10)	0.0054 (9)	0.0252 (10)
C3	0.0446 (13)	0.0445 (12)	0.0315 (11)	0.0167 (10)	0.0029 (9)	0.0216 (10)
C6	0.0442 (13)	0.0484 (13)	0.0360 (12)	0.0294 (11)	0.0081 (10)	0.0192 (10)
C5	0.0595 (16)	0.0646 (16)	0.0396 (13)	0.0418 (14)	0.0199 (12)	0.0194 (12)
C4	0.0612 (16)	0.0555 (15)	0.0277 (11)	0.0264 (13)	0.0098 (10)	0.0156 (10)
P1	0.0221 (3)	0.0226 (3)	0.0254 (3)	0.00653 (18)	0.00367 (18)	0.0144 (2)
O4	0.0277 (7)	0.0282 (7)	0.0405 (8)	0.0032 (6)	−0.0050 (6)	0.0213 (6)
O3	0.0372 (8)	0.0480 (9)	0.0272 (8)	0.0222 (7)	0.0062 (6)	0.0144 (7)
O2	0.0253 (7)	0.0276 (7)	0.0427 (8)	0.0101 (5)	0.0087 (6)	0.0211 (6)
O1	0.0370 (8)	0.0290 (7)	0.0422 (9)	0.0056 (6)	0.0087 (6)	0.0238 (7)

### Geometric parameters (Å, °)

**Table:**

N1—C1	1.452 (2)	C6—C5	1.384 (3)
N1—H1A	0.8900	C6—H6	0.9300
N1—H1B	0.8900	C5—C4	1.379 (4)
N1—H1C	0.8900	C5—H5	0.9300
C2—C3	1.390 (3)	C4—H4	0.9300
C2—C1	1.393 (3)	P1—O1	1.4972 (14)
C2—C7	1.431 (3)	P1—O4	1.5004 (15)
C1—C6	1.368 (3)	P1—O2	1.5537 (14)
N2—C7	1.137 (3)	P1—O3	1.5770 (15)
C3—C4	1.368 (3)	O3—H3A	0.8200
C3—H3	0.9300	O2—H2A	0.8200
			
C1—N1—H1A	109.5	C1—C6—H6	120.1
C1—N1—H1B	109.5	C5—C6—H6	120.1
H1A—N1—H1B	109.5	C4—C5—C6	120.8 (2)
C1—N1—H1C	109.5	C4—C5—H5	119.6
H1A—N1—H1C	109.5	C6—C5—H5	119.6
H1B—N1—H1C	109.5	C3—C4—C5	119.5 (2)
C3—C2—C1	119.88 (18)	C3—C4—H4	120.2
C3—C2—C7	118.03 (18)	C5—C4—H4	120.2
C1—C2—C7	122.07 (18)	O1—P1—O4	113.96 (8)
C6—C1—C2	119.67 (19)	O1—P1—O2	107.38 (8)
C6—C1—N1	119.67 (17)	O4—P1—O2	112.70 (8)
C2—C1—N1	120.64 (16)	O1—P1—O3	109.65 (9)
N2—C7—C2	176.7 (2)	O4—P1—O3	109.18 (9)
C4—C3—C2	120.2 (2)	O2—P1—O3	103.43 (8)
C4—C3—H3	119.9	P1—O3—H3A	109.5
C2—C3—H3	119.9	P1—O2—H2A	109.5
C1—C6—C5	119.9 (2)		
			
C3—C2—C1—C6	−1.2 (3)	C2—C1—C6—C5	0.6 (4)
C7—C2—C1—C6	177.2 (2)	N1—C1—C6—C5	178.9 (2)
C3—C2—C1—N1	−179.47 (18)	C1—C6—C5—C4	0.2 (4)
C7—C2—C1—N1	−1.1 (3)	C2—C3—C4—C5	−0.2 (4)
C1—C2—C3—C4	0.9 (3)	C6—C5—C4—C3	−0.4 (4)
C7—C2—C3—C4	−177.5 (2)		

### Hydrogen-bond geometry (Å, °)

**Table:**

*D*—H···*A*	*D*—H	H···*A*	*D*···*A*	*D*—H···*A*
N1—H1B···O4^i^	0.89	1.93	2.819 (2)	176
N1—H1C···O1^ii^	0.89	1.85	2.723 (2)	166
N1—H1A···O1^iii^	0.89	1.87	2.730 (2)	161
O3—H3A···N2^iv^	0.82	1.98	2.797 (2)	176
O2—H2A···O4^v^	0.82	1.76	2.574 (2)	172

Symmetry codes: (i) *x*−1, *y*, *z*−1; (ii) −*x*+1, −*y*, −*z*+1; (iii) *x*, *y*, *z*−1; (iv) −*x*+2, −*y*, −*z*+1; (v) −*x*+2, −*y*+1, −*z*+2.
